# Annual acknowledgement of manuscript reviewers

**DOI:** 10.1186/1472-6807-13-1

**Published:** 2013-02-19

**Authors:** Simon Harold

**Affiliations:** 1 BioMed Central, Floor 6, 236 Gray's Inn Road, London WC1X 8HB, UK

## Abstract

**Contributing reviewers:**

The editors of *BMC Structural Biology* would like to thank all of our reviewers who have contributed to the journal in Volume 12 (2012).

## 

Emil Alexov

USA

Toshio Ando

Japan

Mariko Ariyoshi

Japan

Shannon Au

Hong Kong

Pradipta Bandyopadhyay

India

Gail Bartlett

UK

Branimri Bertoša

Croatia

Parbati Biswas

India

Titus Boggon

USA

Andrew Bordner

USA

Thomas Boudier

France

Martin Boulanger

Canada

Igor Bronstein

UK

Oliviero Carugo

Austria

Arthur Cederbaum

USA

Marie Chabbert

France

Monica Chagoyen

Spain

Chinpan Chen

Taiwan

S Chakra Chennubhotla

USA

Alexandre de Brevern

France

Gilbert Deleage

France

Doreen Dobritzsch

Sweden

Roberto Dominguez

USA

Francois-Yves Dupradeau

France

Pasqualina D’ursi

Italy

Thomas Edwards

USA

Lindsay Eltis

Canada

Eric Engstrom

USA

Angelo Facchiano

Italy

Mario Fares

Ireland

Natalie Fedorova Abrams

USA

Juan Fernandez-Recio

Spain

Daniel Ferraro

USA

James Ferry

USA

Andras Fiser

USA

Federico Fogolari

Italy

Brian Fox

USA

Shuya Fukai

Japan

Federico Gago

Spain

Zoltán Gáspári

Hungary

Brian Geisbrecht

USA

Fabian Glaser

Israel

Fernando Gonzalez Nilo

Chile

Bruce Goode

USA

Alexander Grishaev

USA

Markus Grütter

Switzerland

Dominik Heider

Germany

Bosco Ho

Australia

Hazel Holden

USA

Shinya Honda

Japan

Kwang Yeon Hwang

South Korea

Atsushi Ishikawa

Japan

Wolfgang Jahnke

Switzerland

David Jeruzalmi

USA

Alkan Kabakcioglu

Turkey

Olga Kalinina

Germany

Richard Kammerer

Switzerland

Natarajan Kannan

USA

Adrian Keatinge-Clay

USA

James Ketudat Cairns

Thailand

Daisuke Kohda

Japan

Joern Krausze

Germany

Susan Krueger

USA

Eduardo Larriba

Spain

Aparna Laskar

India

Roman Laskowski

UK

Shuxing Li

USA

Dennis Livesay

USA

Michael Lobritz

USA

Ray Luo

USA

Natesan Manickam

India

Debora Marks

USA

Kenji Mizuguchi

Japan

Kenji Mizuguchi

UK

Alexey Murzin

UK

Richard Edward Myers

UK

Bhushan Nagar

Canada

Rafael Najmanovich

Canada

Yoji Nakamura

Japan

Kimberly Nelson

USA

Luis Nino

Colombia

Ken Nishikawa

Japan

Teresa Olczak

Poland

Natalia Osna

USA

Motonori Ota

Japan

Anna Panchenko

USA

Florencio Pazos

Spain

Alex Perryman

USA

Eskild Petersen

Denmark

Michael Pikaart

USA

Igor Polikarpov

Brazil

Shalom Rackovsky

USA

Aaron Rashotte

USA

Charles Robert

France

Ernesto Roman

Argentina

Vicente Rubio

Spain

Gunter Schneider

Sweden

Jana Selent

Spain

Trevor Sewell

South Africa

Gary Shaw

Canada

X Shen

China

Armando Solis

USA

Patricia Soto

USA

Thomas Steinbrecher

Germany

Xiaolin Sun

New Zealand

Autumn Sutherlin

USA

Michel Sylvestre

Canada

Eric Toth

USA

Pierre Tuffery

France

Nurcan Tuncbag

USA

Takayuki Uchihashi

Japan

Focco van den Akker

USA

Mauno Vihinen

Sweden

Dierk Wanke

Germany

Dongqing Wei

China

Martin Weigt

France

Dek Woolfson

UK

Jinbo Xu

USA

Ting Xu

USA

Zhe Yang

USA

Chen Yanover

USA

Guo Rong Yin

China

Bojan Zagrovic

Croatia

Snezana Zaric

Serbia

